# High expression of serine protease 2 (PRSS2) associated with invasion, metastasis, and proliferation in gastric cancer

**DOI:** 10.18632/aging.204604

**Published:** 2023-03-23

**Authors:** Haifeng Qin, Shushu Zhang, Linling Shen, Chenjian Mao, Guangyu Gao, Hui Wang

**Affiliations:** 1 Department of Nuclear Medicine, First People’s Hospital of Kunshan, Kunshan, Suzhou 215004, Jiangsu Province, China; 2 Department of Oncology, The Second Affiliated Hospital of Soochow University, Suzhou 215004, Jiangsu Province, China; 3 Department of Ultrasound, The Second Affiliated Hospital of Soochow University, Suzhou 215004, Jiangsu Province, China

**Keywords:** PRSS2, gastric cancer, GEO, CCK-8, qPCR

## Abstract

Background: Accumulating evidence indicates that the occurrence and development of tumors are related to the activation of oncogenes and the inactivation of tumor suppressor genes caused by epigenetic mechanisms. However, the function of serine protease 2 (PRSS2) in gastric cancer (GC) is still unknown. Our study aimed to find a regulation network involved in GC.

Methods: The mRNA data (GSE158662 and GSE194261) of GC and normal tissues were downloaded from the Gene Expression Omnibus (GEO) dataset. Differential expression analysis was performed using R software, and Gene Ontology (GO) analysis and Kyoto Encyclopedia of Genes and Genomes (KEGG) pathway analysis was conducted by using Xiantao software. Besides, we used Quantitative Real-time PCR (qPCR) to verify our conclusions. After gene knockdown, cell migration and CCK-8 experiment were carried out to detect the effect of gene on cell proliferation and invasion.

Results: Totally, 412 differentially expressed genes (DEGs) were identified from GSE158662 and 94 DEGs were identified from GSE196261. Km-plot database results indicated that PRSS2 exhibited high diagnosis worth for GC. Gene functional annotation enrichment analysis revealed that these hub mRNAs were mainly taken part in the process of tumorigenesis and development. Besides, vitro experiments showed that down-regulation of PRSS2 gene reduced the proliferation and invasion ability of GC cells.

Conclusions: Our results indicated that PRSS2 may play vital roles in the carcinogenesis and progression of GC and can be potential biomarkers for patients with GC.

## INTRODUCTION

Gastric carcinoma (GC) is a malignant carcinoma derived from gastric mucosal epithelium, ranking first in the incidence of various malignant tumors in the world. The prognosis of GC is associated with its pathological stage, location, and histological type. The morbidity and mortality of GC are very worse. The total OS of patients with advanced GC is only one year [1, 2]. This disappointing survival outcome is mainly due to the inherent biological invasiveness of gastric cancer and the relatively weak response to existing treatment. Chemotherapy for advanced gastric cancer mostly adopts fluorouracil-based combination chemotherapy for first-line and second-line treatment, but the efficacy is low. The objective response rate (ORR) of first-line treatment is only 35~55%, the progression free survival (PFS) is only 4~10 months, and the median overall survival (mOS) is only 9~14 months, with severe side effects [3, 4].

Among the monoclonal antibodies targeting pro-grammed cell death receptor 1 (PD-1)/programmed cell death ligand 1 (PD-L1), nivolumab, avelumab, and pembrolizumab have been most studied in the 3-line treatment of gastric cancer [5, 6]. Nivolumab, as an all human IgG4 monoclonal antibody, evaluated a cohort of 493 advanced GC Asian patients (regardless of programmed cell death ligand 1 status) who received at least two systematic regimens in a phase III clinical study. The results showed that immunotherapy had better long-term efficacy and survival benefits [7, 8]. The clinical research results of KEYNOTE-059 prompted Food and Drug Administration to approve pembrolizumab as a third line treatment scheme for patients with advanced stage at the gastroesophageal junction with programmed cell death ligand 1 CPS 1 expression [8, 9]. Avelumab is a human anti programmed cell death ligand 1 IgG1 monoclonal antibody approved for use in other tumors. In 2018, in a phase III clinical trial to explore the effectiveness and safety of avelumab, avelumab and doctors selected systematic treatment as the third line treatment for patients with advanced GC in 371 randomized cohort patients. The results showed that immunotherapy had fewer treatment-related adverse reactions than systemic therapy [10].

In this study, based on GSE158662 and GSE194261 datasets, differentially expressed miRNAs and mRNAs were identified by integrating multiple bioinformatics analysis methods. Our research serves as an important resource to further analyze the mechanisms of GC progression.

## RESULTS

### Identification of DEGs

**Figure 1:**
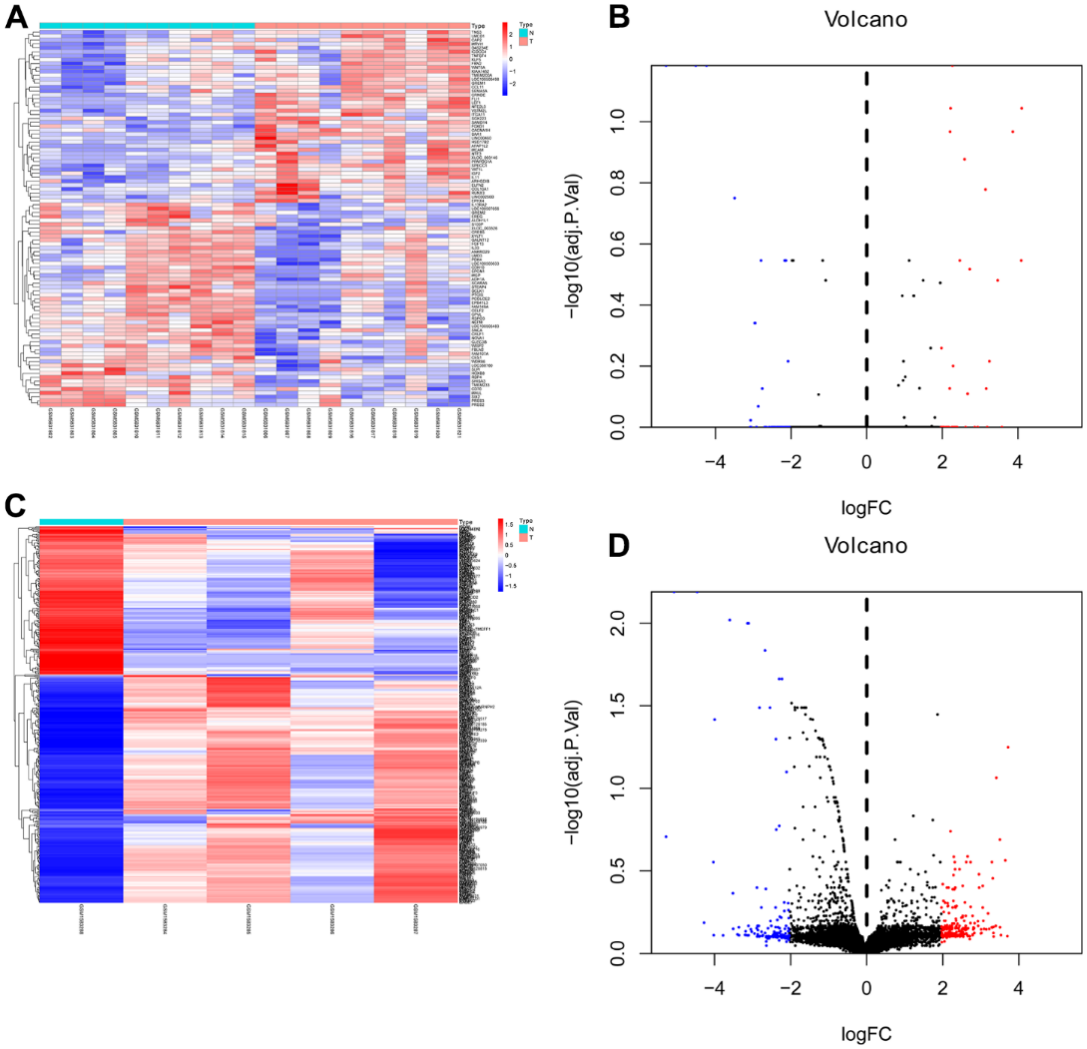
**Heat map and volcano map of differentially expressed genes of GSE158662 and GSE194261.** (**A**, **B**) Heat map and volcano map of DEGs in GSE158662. (**C**, **D**) Heat map and volcano map of DEGs in GSE194261.

R software was used to analyze the DEGs from the GSE158662 and GSE194261. After screening, 412 DEGs such as STYK1, LINC00488, IFNA22P, FEZF1, NR4A1, and C15orf34 were obtained from GSE158662. By the same method, 96 DEGs such as PCOLCE2, SHISA3, SPECC1, IL13RA2, SAMD14, and SAA1 were obtained from GSE194261 (Figure 1).

### Gene ontology and KEGG enrichment analysis

**Figure 2:**
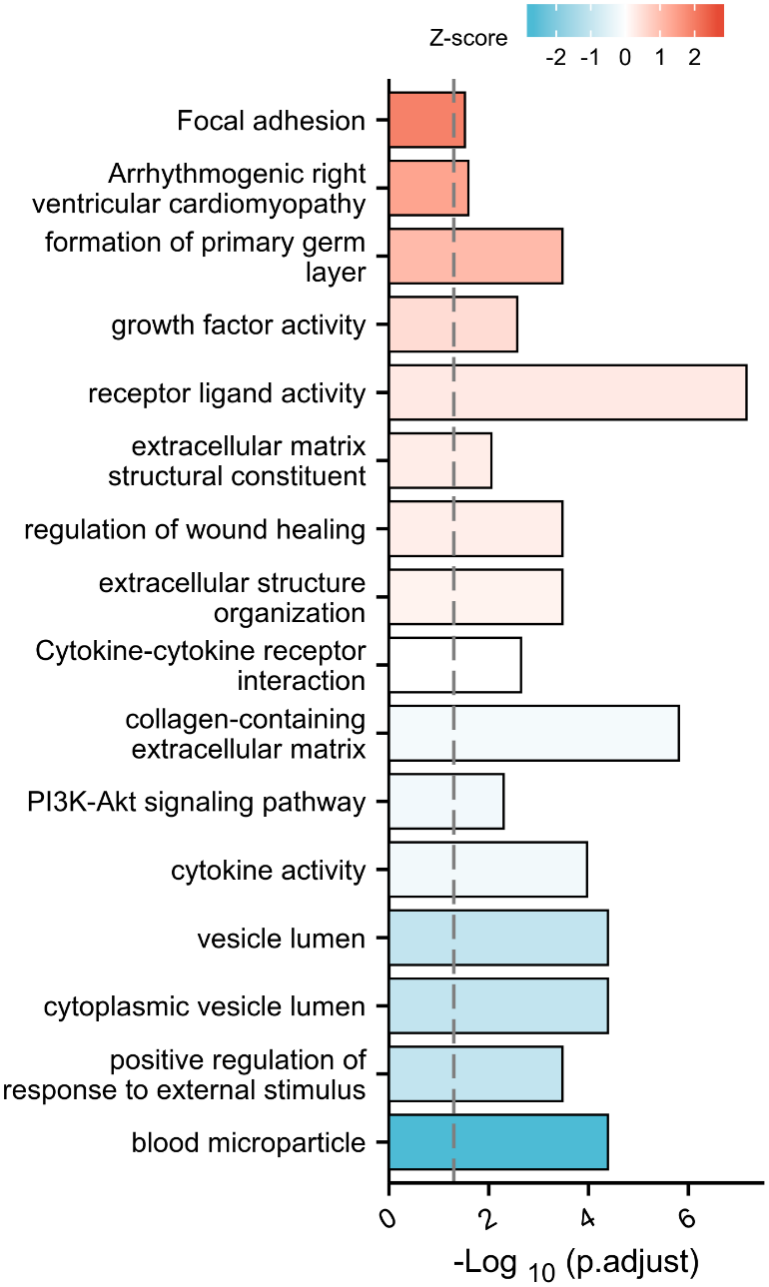
Gene ontology and KEGG enrichment.

Xiantao software is an independent software tool mainly used for gene and protein function enrichment and interaction network analysis. GO and KEGG analysis showed that DEGs had the most uniquely enriched terms for blood microparticle, positive regulation of response to external stimulus, cytoplasmic vesicle lumen, vesicle lumen, cytokine activity, PI3K-Akt signaling pathway, collagen-containing extracellular matrix, Cytokine-cytokine receptor interaction, extracellular structure organization, regulation of wound healing, extracellular matrix structural constituent, receptor ligand activity, growth factor activity, formation of primary germ layer, Arrhythmogenic right ventricular cardiomyopathy, and Focal adhesion (Figure 2).

### The association with genes expression and GC overall survival

**Figure 3:**
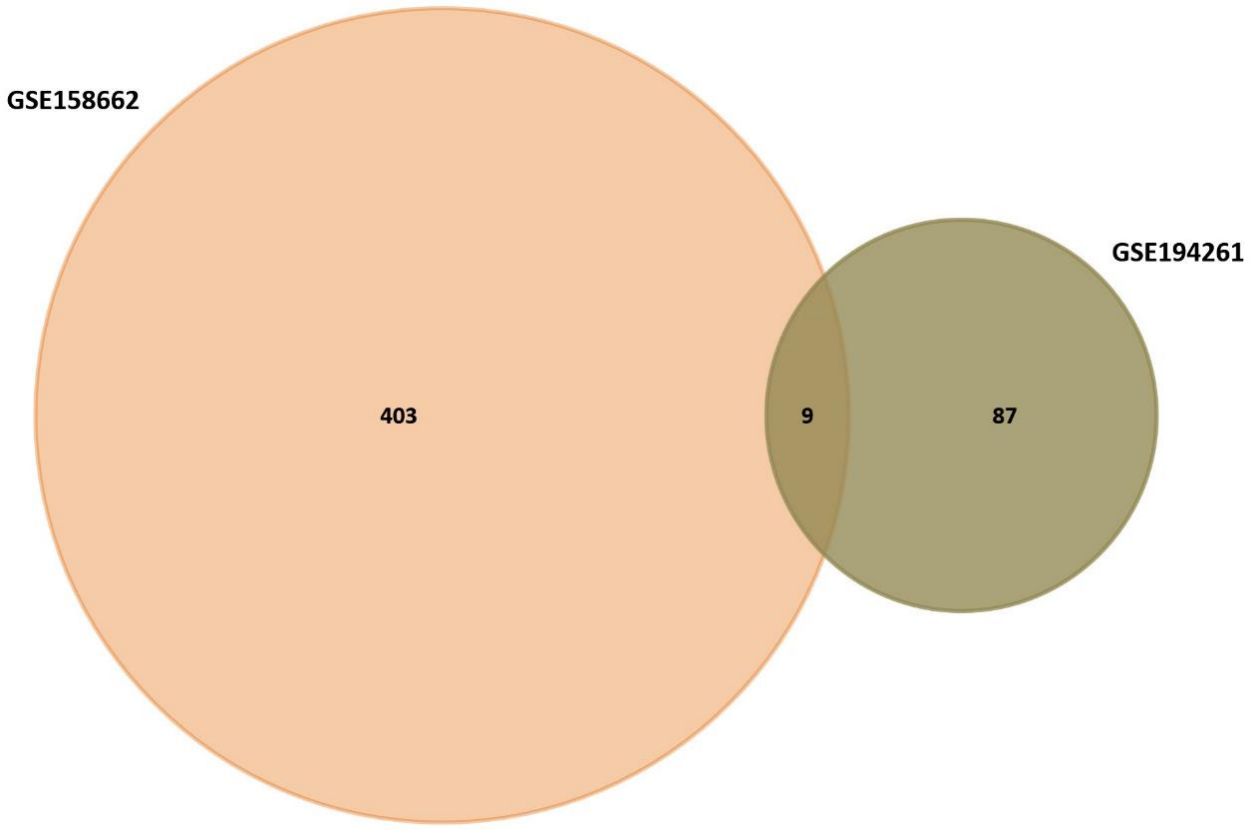
Venn diagram of GSE158662 and GSE194261.

**Figure 4:**
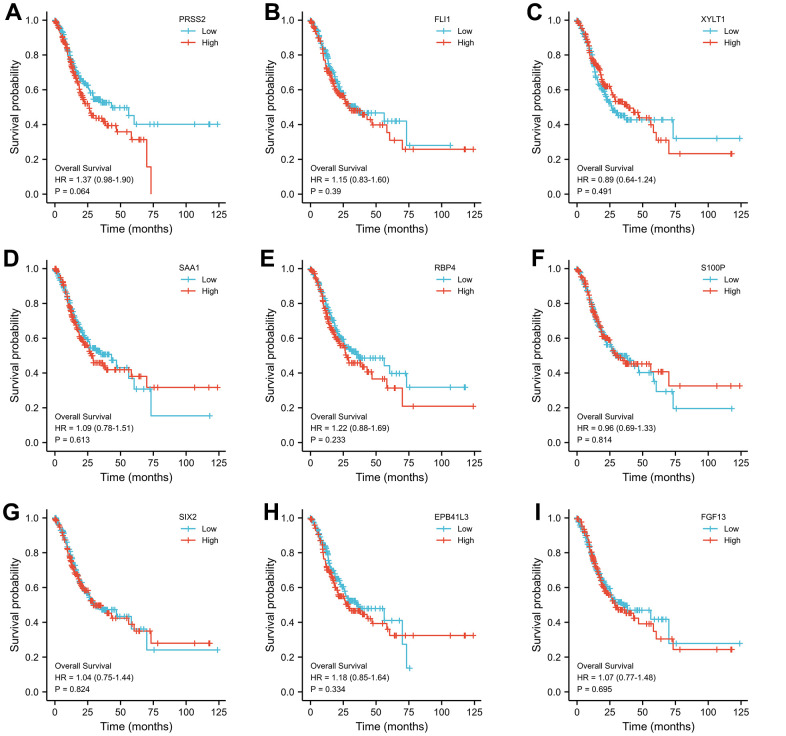
**The relationship between the expression level of selected genes and overall survival of gastric cancer patients.** (**A**–**I**) Relationship between various molecules including PRSS2, SIX2, FGF13 and survival of gastric cancer patients.

Through Venn diagram, 9 common differentially expressed genes were selected for further study (Figure 3). The prognostic value of the 9 common differentially expressed genes was evaluated through the KaplanMeier plotter database. The results showed that PRSS2 were related to gastric cancer OS (Figure 4). Therefore, we chose PRSS2 for further study.

### Immune infiltration analysis

**Figure 5:**
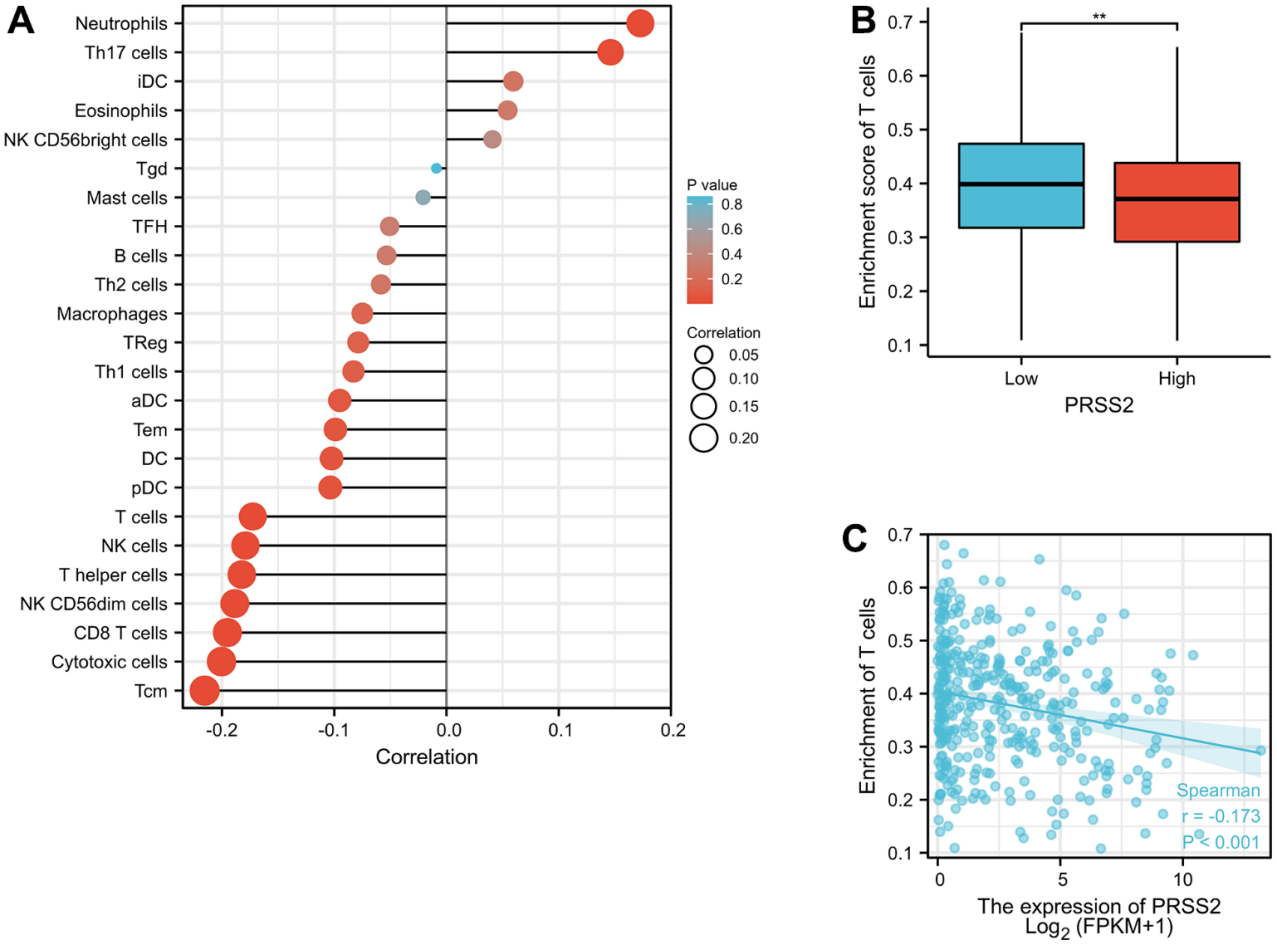
**The relationship between PRSS2 and immune infiltration.** (**A**) Immunocyte infiltration and enrichment analysis. (**B**, **C**) High expression of PRSS2 in gastric cancer is associated with less T cell infiltration.

Immune infiltration analysis is to use transcriptome or other histologic data to estimate the score of immune cells in the tissue through algorithms. It can be used to analyze whether the immune cell scores of each sample in the high and low expression groups of a single molecule are statistically different. By utilizing Xiantao software, we found that PRSS2 was mostly related to Neutrophils, Th17 cells, Cytotoxic cells, and Tcm. High expression of PRSS2 in gastric cancer is associated with less T cell infiltration (Figure 5).

### The relationship between clinical characteristics and PRSS2 expression of gastric cancer

Xiantao bioinformatics tool encapsulates and simplifies some common analysis and visualization functions in R language, realizes online analysis and visualization through front-end technology, quickly solves common statistical analysis and visualization contents in one stop, and provides online jigsaw puzzles to directly output publication level whole pictures. It can easily and quickly complete the visualization of analysis and publication level images, with rich and comprehensive parameters, personalized output results, and provide data and image downloads in various formats at the same time. In addition, it can also adjust color matching, statistical methods, text position, color, line color, style, thickness, transparency, title text, illustration, style and other contents. Clinical and gene expression information of patients with gastric cancer were obtained from the Xiantao software including TNM stage, age, OS event, and primary therapy outcome (Table 1). TNM stage is the staging standard of the International Federation of Obstetrics and gynecology for gynecological tumors, including cervical cancer, ovarian cancer, fallopian tube and peritoneal cancer. According to TNM's staging standard, tumors are usually divided into four stages. The results indicated that TNM stage (P<0.05) and Primary therapy outcome (P<0.05) was associated with the overall survival of patients with gastric cancer (Table 2).

**Table 1:** Relationship between the expression level of PRSS2 and clinical features.

**Characteristic**	**Low expression of PRSS2**	**High expression of PRSS2**	**p**
n	187	188	
T stage, n (%)			0.277
T1	8 (2.2%)	11 (3%)	
T2	37 (10.1%)	43 (11.7%)	
T3	80 (21.8%)	88 (24%)	
T4	58 (15.8%)	42 (11.4%)	
N stage, n (%)			0.672
N0	60 (16.8%)	51 (14.3%)	
N1	45 (12.6%)	52 (14.6%)	
N2	36 (10.1%)	39 (10.9%)	
N3	39 (10.9%)	35 (9.8%)	
M stage, n (%)			0.382
M0	169 (47.6%)	161 (45.4%)	
M1	10 (2.8%)	15 (4.2%)	
Primary therapy outcome, n (%)			0.961
PD	32 (10.1%)	33 (10.4%)	
SD	8 (2.5%)	9 (2.8%)	
PR	2 (0.6%)	2 (0.6%)	
CR	120 (37.9%)	111 (35%)	
OS event, n (%)			0.421
Alive	118 (31.5%)	110 (29.3%)	
Dead	69 (18.4%)	78 (20.8%)	
Age, median (IQR)	67 (58, 73)	67.5 (59, 73.75)	0.452

**Table 2:** The association between OS and the expression level of PRSS2 was studied by univariate and multivariate Cox regression.

**Characteristics**	**Total(N)**	**Univariate analysis**			**Multivariate analysis**	
		**Hazard ratio (95% CI)**	**P value**		**Hazard ratio (95% CI)**	**P value**
PRSS2	370	1.751 (1.190-1.915)	**0.001**			
T stage	362					
T1	18	Reference				
T3&T4&T2	344	8.829 (1.234-63.151)	**0.030**		22692221.327 (0.000-Inf)	0.994
N stage	352					
N0	107	Reference				
N1&N2&N3	245	1.925 (1.264-2.931)	**0.002**		1.398 (0.860-2.273)	0.177
M stage	352					
M0	327	Reference				
M1	25	2.254 (1.295-3.924)	**0.004**		1.691 (0.874-3.274)	0.119
Primary therapy outcome	313					
SD&PR&CR	249	Reference				
PD	64	4.147 (2.843-6.047)	**<0.001**		3.532 (2.386-5.229)	**<0.001**

### Knockdown of PRSS2 suppresses GC cell growth

**Figure 6:**
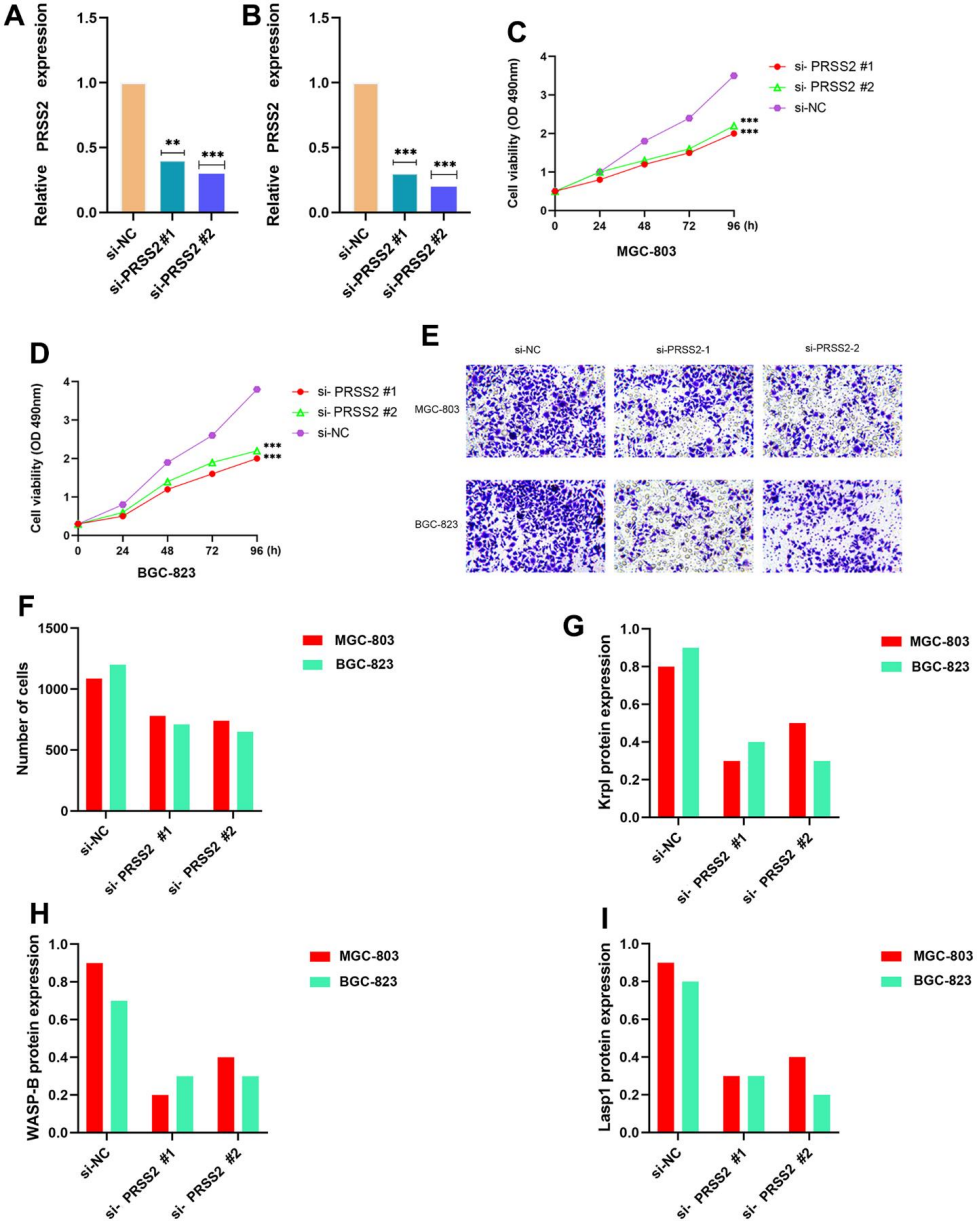
**Effects of PRSS2 knockdown on GC cell viability and migratory capacity *in vitro*.** (**A**, **B**) PRSS2 expression in MGC-803 and BGC-823 cells transfected with negative control siRNA (si-NC) or siRNAs targeting PRSS2 (si-PRSS2 #1 and #2) for 48h n=3 for each group. (**C**, **D**) Cell viability was assessed using a CCK-8 assay in MGC-803 and BGC-823 cells transfected with si-NC or si-PRSS2 #1 and #2 for 48h n=6 for each group. (**E**, **F**) Transwell invasion assay was performed to determine the invasion ability of si-PRSS2-transfected MGC-803 and BGC-823 cells for 48h n=3 for each group. (**G**–**I**) qPCR detection of invasive pseudopod-related proteins Krp1, WASP-B, and Lasp1. n = 3. ^*^P < 0:05, ^**^P < 0:01, and ^***^P < 0:001 indicate significant difference.

To investigate the potential biological function of PRSS2 in GC cells, we down-regulated the expression of PRSS2 in MGC-803 and BGC-823 cells by transfecting two independent siRNAs (#1 and #2, respectively). Both siRNAs significantly reduced the expression of PRSS2 (Figure 6A, 6B). The results of CCK-8 assay showed that the proliferation ability of MGC-803 and BGC-823 cells decreased after down-regulation of PRSS2 (Figure 6C, 6D). Through Transwell experiments, we further confirmed that silencing of PRSS2 inhibited the invasion ability of MGC-803 and BGC-823 cells (Figure 6E, 6F). PRSS2 knockdown also obviously suppressed the activity of invasive pseudopod-related proteins Krp1, WASP-B, and Lasp1 (P < 0.001) (Figure 6G–6I).

## DISCUSSION

Gastric cancer is one of the most common malignant tumors in China, with the highest incidence rate of digestive tract tumors. About 170000 people die of gastric cancer every year, which seriously threatens the health of the population. Gastric cancer can occur at any age, but between the ages of 40 and 60, more men than women, about 3:1. Cancer can occur in any part of the stomach, but it is most common in the gastric antrum, especially the small curvature of the stomach. The average life expectancy of untreated people is about 13 months [11, 12]. In our study, GSE81873 and GSE27651 were obtained in the Gene Expression Omnibus database. KEGG analysis demonstrated that DEGs were mainly enriched in 6 pathways as Arrhythmogenic right ventricular cardiomyopathy, Focal adhesion, Cell adhesion molecules (CAMs), PI3K-Akt signaling pathway, and Cytokine-cytokine receptor interaction, which were indicated to influence development and recurrence [13–15]. Cell adhesion molecules (CAM) are proteins located on the surface of cells and participate in the binding process called cell adhesion to other cells or extracellular matrix (ECM) [16]. In essence, cell adhesion molecules help cells adhere to each other and adhere to the environment. These proteins are usually transmembrane and consist of three domains: the interaction with cytoskeleton, transmembrane domain (cell surface) and extracellular domain in the intracellular domain, or with other cell adhesion molecules of the same type (homophilic bond), or with other cell adhesion molecules or extracellular matrix (heterophilic bond) [17]. Most cell adhesion molecules (CAM) belong to four protein families: immunoglobulin superfamily (IGSF-CAM), integrin, cadherin and selectin. The classification system involves the difference between non-calcium-dependent cell adhesion molecules and calcium-dependent cell adhesion molecules [18]. E, l and P respectively represent the endothelial cells, leukocytes and platelets that were originally isolated to obtain these three selectins. L-selectin was first found as a homing receptor on lymphocytes, and later found to be expressed on all kinds of leukocytes; P-selectin exists in the storage granules of platelets and endothelial cells, and can be transported to the cell surface within a few minutes after cell activation; E-selectin, which exists in endothelial cells, is synthesized and transported to the cell surface after cell activation. The membranous region of each member has high homology and structural similarity, but there is no homology between the transmembrane region and the cytoplasmic region. The ligands recognized by selectin are all oligosaccharide groups, and the ligands found so far are molecules with sialylated Lewis oligosaccharides or similar structures. An oligosaccharide group can exist on a variety of glycoproteins and glycolipids, and is distributed on a variety of cell surfaces, so selectin molecular ligands are widely distributed in the body [19]. Selectin, which is distributed in leukocytes, endothelial cells and platelets, mediates the recognition and combination of leukocytes and endothelial cells, participates in the process of leukocytes crossing blood vessels into inflammatory tissues and lymphocyte homing and recycling, and plays a vital role in plenty of physiological or pathological activities such as glomerulonephritis, multiple sclerosis, coagulation, tumor metastasis, insulin-dependent diabetes [20].

Trypsin is a serine protease, which plays an important role in the development of tumor. Trypsin family members include three isoenzymes (serine protease 1, serine protease 2 and serine protease 2), of which serine protease 2 (PRSS2) is a neutral serine protease [21, 22]. PRSS2 was first found in the pancreas and plays a role in food digestion. serine protease 2 can reduce trypsin activity and protect chronic pancreatitis [23]. The results showed that PRSS2, as an activator of matrix metalloproteinase (MMP), caused the degradation of peripheral collagen by activating interstitial collagenase. serine protease 2 has been proved to be associated with the development of tongue carcinoma, pancreatic carcinoma, colorectal carcinoma and ovarian carcinoma [24–26]. The high expression of serine protease 2 in pancreatic carcinoma is related to T phase. In breast cancer, the expression of serine protease 2 induced by C-terminal-binding protein 1 is associated with tumor progression and metastasis [27]. Previous studies have shown that serine protease 2 is up-regulated in gastric cancer tissue and is an independent prognostic indicator after gastric cancer surgery [28]. However, the expression of serine protease 2 in blood samples of gastric cancer patients and healthy people needs further analysis to evaluate its early clinical diagnostic value for gastric cancer. Tumor invasion and metastasis is a complex and continuous process of interaction between tumor cells and host cells [29]. serine protease 2 is a serine protease that can degrade extracellular matrix, activate other proteases related to cancer invasion, and promote the development of gastric cancer [30]. The cascade of protein hydrolysis is complex, and the activation of one protease will lead to the increase of the activities of several downstream proteases [31]. Related mechanism studies also showed that protease-activated receptor 2 activated trypsin through proteolysis, thereby activating intracellular signals, such as MAPK/ERK, which are closely related to cell growth, differentiation and survival. In addition, serine protease 2 can be used as the downstream target of activated K-RAS oncogene to promote the occurrence of pancreatic cancer [32, 33].

PASS2 play key roles in various tumors. Our study indicated that plenty of DEGs took part in the development of gastric cancer. Therefore, suppression of PASS2 may have important worth in GC patients.

## CONCLUSIONS

In our research, we utilized bioinformatics and experimental methods were used to analyze the differentially expressed genes in GC and normal tissues. PASS2 were selected as latent biomarkers of GC. However, more cell experiments were needed to prove it.

## MATERIALS AND METHODS

### Database screening

NCBI (National Center for Biotechnology Information) is the National Biotechnology Information Center of the United States. He can search and analyze molecular databases and biomedical articles around the world, and develop software tools for analyzing genomic data and disseminating biomedical information. GSE158662 and GSE194261 were obtained from the GEO database.

### Difference analysis

R language is one of the current mainstream analysis software, which has powerful data processing and analysis functions, including: basic sequence analysis, molecular evolution and comparative genomics; Protein structure comparison and prediction; Computer aided drug design, etc. Bioconductor is based on the R language environment, which is used for the annotation, processing, analysis of biological information data and the collection of visual tools. It is composed of a series of R extension packages. Limma and ggplot2 R package was utilized to identify DEGs and DEMs. |log2FC|> 2 and FDR<0.05 were set as standards.

### GO and KEGG pathway analysis

The gene ontology (GO) project aims to obtain consistent results in the functional description of gene products in various databases. The project started in 1988 and integrated three modal biology databases: fly enzyme (*Drosophila melanogaster*), T yeast genome database (SGD) and mouse genome database (MGD). Since then, Go has continued to grow and now has dozens of animal, plant and microbial databases. The Go definition law has been used in many cooperative databases, which makes the queries in these databases highly consistent. This definition language has multiple structures, so it can be queried to different degrees. For example, GO can be used to interrogate and transduce the signal related gene products in the mouse genome, and can also find various bio-derived receptor tyrosine kinases. This structure allows knowledge about the characteristics of this genetic product to be increased at different levels. KEGG (Kyoto Encyclopedia of genes and genes) database is a database that systematically analyzes gene functions and links genome information and functional information, including metabolic pathway database, hierarchical classification database, gene database, genome database, etc. KEGG's pathway database is the most widely used public database of metabolic pathways. Xiantao software was utilized for further study.

### The association between DEGs and clinical feathers in TCGA

Km-plot was used to perform survival analysis. The current TCGA data is included in GDC, including statistical data. As of 2019, TCGA has covered 68 human tissues/organs, 45 articles and 33549 patients, with a total of 365463 records. Explore: It allows users to explore data using various case, gene and mutation filters. Analysis: allows users to compare the functions of different queues. These queues can be generated through existing filters (for example, lung cancer patients) or personalized selection. Repository: Here, users can view the data files that can be downloaded from GDC, and apply the file/CAS filter to optimize the search. Body contour: the main page displays the human anatomy contour.

### Immune infiltration

Xiantao bioinformatics tool encapsulates and simplifies some common analysis and visualization functions in R language, realizes online analysis and visualization through front-end technology, quickly solves common statistical analysis and visualization contents in one stop, and provides online jigsaw puzzles to directly output publication level whole pictures. It can easily and quickly complete the visualization of analysis and publication level images, with rich and comprehensive parameters, personalized output results, and provide data and image downloads in various formats at the same time. In addition, it can also adjust color matching, statistical methods, text position, color, line color, style, thickness, transparency, title text, illustration, style and other contents. It uses transcriptome or other omics data to estimate the score of immune cells in the tissue through algorithms.

### Cell culture and transfection

GC-derived cell lines (MGC-803 and BGC-823) and normal human gastric surface epithelial cell lines (HGSE) were obtained from the Institute of Cell Biology, Chinese Academy of Sciences (Shanghai, China). All cells were cultured in Roswell Park Memorial Institute-1640 (RPMI-1640) medium (HyClone, South Logan, UT, USA) containing 10% fetal bovine serum (FBS; Gibco, Rockville, MD, USA), and maintained in a 37° C, 5% CO2 incubator. MGC-803 and BGC-823 cells were transfected with Lipofectamine 2000 (Invitrogen) and PRSS2 or negative control siRNA(si-NC) were transfected according to the manufacturer's instructions. The sequences of siRNA are si-PRSS2-1: GGTAAGGAGCATCGATCAC, si-PRSS2-2: GGTAAAGGTCACTAGCCAA; si-NC: ACGUGACACGUUCGGTCA.

### Cell viability and invasion assays

Preparation of matrix free adhesive Transwell cell:Coated basement membrane: Coat the upper chamber surface of the bottom membrane of Transwell cell with 50mg/L Matrigel 1:8 diluent, and air dry at 4° C. Hydrated basement membrane: Suck out the residual liquid in the culture plate, add 50 ul of serum-free culture solution containing 10 g/LBSA into each well, 37° C, 30 min. Preparation of cell suspension: Before preparing the cell suspension, the cells can be starved for 12-24 hours by removing the serum to further remove the influence of serum. Digestive cells were centrifuged and discarded after digestion, washed with PBS for 1-2 times, and resuspended with serum free medium containing BSA. At least, it is necessary to ensure that there is a certain amount of cells in the upper room when collecting samples. Inoculated cells: Take 100-200 μ l of cell suspension and add it into the chamber. Add 500 μ l culture medium containing FBS or chemokine into the lower chamber of the 24-well plate. Cultured cells: cultured for 12-48h (mainly depending on the invasive ability of cancer cells). In addition to considering the invasiveness of cells, the effect of processing factors on the number of cells should not be ignored.

### Availability of data and materials

The datasets used and/or analyzed during the current study are available from the corresponding author on reasonable request.
